# Oral Health in Early Childhood: A Cross-Sectional Study on Maternal Knowledge, Attitude and Practice

**DOI:** 10.7759/cureus.89052

**Published:** 2025-07-30

**Authors:** Pranamee Barua, Prabhat Kumar, Bhaskar Das, Afshan Khan, Sampanna Kalita, Anindita Talukdar, Seema Gupta

**Affiliations:** 1 Department of Pedodontics and Preventive Dentistry, Regional Dental College, Guwahati, IND; 2 Department of Pedodontics and Preventive Dentistry, Dental College Azamgarh, Azamgarh, IND; 3 Department of Dentistry, Diphu Medical College and Hospital, Diphu, IND; 4 Department of Orthodontics, Kothiwal Dental College and Research Centre, Moradabad, IND

**Keywords:** awareness, early childhood, knowledge, mothers, oral health, practice

## Abstract

Introduction: Oral health in early childhood is critical for long-term well-being; however, parental knowledge, attitude and practice (KAP) significantly influence children’s oral health outcomes. This hospital-based cross-sectional study aimed to evaluate the KAP of mothers regarding their children’s oral health and to inform strategies for reducing the prevalence of oral diseases. The objectives were to assess maternal knowledge of oral health, attitudes toward dental check-ups and hygiene practices, oral health-related behaviors, and the impact of education and income on KAP, providing insights for targeted interventions.

Materials and methods: This cross-sectional study was conducted in the Department of Pedodontics and Preventive Dentistry. This study targeted 300 mothers of children aged 2-6 years. The questionnaire in English, Hindi, and Assamese, validated through a pilot study (Cronbach’s alpha=0.81), included three sections: knowledge (K1-K10), attitude (A1-A10), and practice (P1-P10). The scoring criteria followed a modified Likert scale, with correct responses scored as 1 and incorrect or “don’t know” responses scored as 0. Statistical analyses included chi-square goodness-of-fit test, one-way analysis of variance, and Pearson’s correlation, with a significance threshold of p<0.05.

Results: Most mothers demonstrated strong knowledge, with 290 (97%) recognizing the importance of twice-daily brushing and 250 (83%) being aware of the caries risk in children aged two years. However, only 165 (55%) participants were aware of their correct weaning age. Attitudes were positive, with 275 (92%) valuing balanced diets and 280 (93%) seeking dental health knowledge. However, only 150 (50%) acknowledged bacterial transmission risks. The practices were robust, with 280 (93%) assisting with brushing, 280 (93%) offering plain water after feeding, and 85 (28%) pre-chewing food. Higher education and income significantly improved KAP scores (p<0.05), with education (r=0.65) and oral health education (r=0.78) strongly influencing practices.

Conclusion: Although maternal KAP was strong in the study, socioeconomic disparities and specific knowledge gaps highlighted the need for community-based and culturally tailored oral health programs to enhance practices and reduce childhood oral diseases.

## Introduction

Oral health is a vital component of overall health and well-being in children, influencing their nutrition, speech, self-esteem, and quality of life [1]. Despite its importance, oral health is frequently neglected in India, particularly in areas with limited awareness and access to dental care [2]. In India, dental caries and periodontal diseases are prevalent among children, with studies indicating that over 52% of children experience dental caries during adolescence [3]. Parental knowledge, attitude and practice (KAP) regarding oral health significantly shape children’s oral health outcomes, making it critical to investigate these factors to inform effective interventions for improving pediatric oral health [4].

Parents, as primary caregivers, are central to establishing their children’s oral hygiene habits, facilitating access to dental care, and making informed health-related decisions [5]. Knowledge of oral health, including the causes of dental diseases, preventive measures, and the importance of early intervention, directly impacts parental attitudes and behaviors [5]. Positive attitudes such as valuing regular dental check-ups or prioritizing oral hygiene often translate into proactive practices such as supervising children’s brushing or limiting sugary diets [6]. Conversely, misconceptions or lack of awareness can lead to neglect of oral health, delayed treatment, or reliance on ineffective traditional practices, exacerbating dental issues in children [7].

Socioeconomic factors, education levels, and access to healthcare contribute to variations in oral health awareness and practices among parents [8]. Urban populations may have better access to dental services; however, cultural beliefs, time constraints, and financial barriers can impede optimal practices. In rural areas, the limited availability of dental professionals or specialists and low awareness of preventive care aggravate this problem [9]. Previous studies have highlighted gaps in parental knowledge, such as underestimating the importance of primary teeth or misunderstanding the role of fluoride in preventing caries [10,11]. These gaps often result in poor oral hygiene practices and infrequent dental visits, contributing to a high prevalence of oral diseases among children.

This hospital-based questionnaire study aimed to evaluate the KAP of parents regarding their children’s oral health to inform strategies for reducing the prevalence of oral diseases among children aged 2-6 years. The objectives were to determine the extent of parents’ knowledge about oral health, assess parents’ attitudes toward the importance of regular dental check-ups and oral hygiene practices, examine parents’ oral health practices, and identify the effects of mothers’ education and income on their children’s oral health, thereby providing insights for developing targeted interventions and community-based oral health programs.

## Materials and methods

Study design and setting

This hospital-based cross-sectional study was conducted in the Department of Pedodontics and Preventive Dentistry at Regional Dental College (RDC), Guwahati, India. Ethical approval was obtained from the Institutional Ethical Committee (RDC/29/2011/2682, dated 29-08-2023) before study initiation. All participants provided written informed consent after being briefed on the study’s objectives, procedures, and right to withdraw at any time without consequences. The confidentiality of participant data was maintained throughout the study. This study was conducted in accordance with the principles of the Declaration of Helsinki.

Inclusion and exclusion criteria

The study included mothers or primary caregivers of children aged 2-6 years who had visited the department. Participants were required to be actively involved in the daily care of their children. Parents of children with special needs and those who did not give their consent were excluded to ensure sample homogeneity.

Sample size estimation

The sample size for the present study was estimated using calculator.net. Considering the prevalence of oral health awareness in mothers as 65% in a study by Suresh et al. [12] and applying the formula n=Z2 × p (1-p)/E2, a sample of 248 participants would suffice 90% power and 5% confidence (alpha error) for the study. A 20% non-response rate was added to the sample, leading to the inclusion of 300 participants in the study. The sample size calculation was based solely on the overall prevalence of oral health awareness (65%) and did not account for subgroup analyses by income or education strata.

Questionnaire design

The questionnaire was developed by the principal investigator, fluent in English, Hindi and Assamese, to ensure cultural and linguistic appropriateness. In the Kamrup (Metro) district of Assam, where multiple languages and dialects are spoken, the questionnaire was developed in English, Assamese, and Hindi, and administered by personnel fluent in these three dialects to ensure cultural and linguistic appropriateness. The instrument included binary (yes/no) questions and rating questions using a modified Likert scale (“yes,” “no,” or “don’t know”). The questionnaire was available in English, Hindi and Assamese to accommodate the participants’ language preferences. For brevity, only the English version is included here (provided in appendices as tables). Translations were prepared and validated during pretesting.

Instrument pretesting

The questionnaire underwent rigorous pre-testing to ensure its reliability and content validity. A pilot study was conducted with a convenience sample of 30 parents who brought their children to the department. The pretesting phase aimed to evaluate the clarity, comprehensibility, and cultural relevance of the questions and the feasibility of administration. Participants in the pilot study provided feedback on question wording, response options, and overall ease of understanding, which were used to refine the questionnaire. To assess reliability, Cronbach’s alpha was computed for the pilot data, confirming a value of 0.81 and indicating strong internal consistency. Test-retest reliability was also evaluated by administering the questionnaire to the same 30 participants after a two-week interval, and the correlation coefficient for repeated responses was calculated to be 0.82, demonstrating stable and consistent responses over time. Content validity was ensured by involving a panel of experts, including two pediatric dentists and one public health researcher, who reviewed the questionnaire for relevance, accuracy, and alignment with the study objectives. The panel assessed whether the questions adequately covered the key domains of KAP related to pediatric oral health. Modifications based on expert feedback included simplifying complex terms and adding questions to address local oral health practices. The final questionnaire was deemed to be clear, relevant, and suitable for the target population.

Scoring methods and outcome assessment

This hospital-based study assessed parental KAP regarding their children’s oral health using a structured questionnaire. The questionnaire comprised three sections: knowledge (K1-K10), attitude (A1-A10), and practice (P1-P10), each containing 10 questions to quantify parental understanding, beliefs, and behaviors, with a maximum score of 10 points per section. Knowledge was evaluated through questions on dental caries causes, fluoride’s role, and preventive measures, with correct responses scored as 1 and incorrect or “don’t know” responses scored as 0; a score ≥6 points (≥60%) indicated good knowledge. Attitudes were assessed using a modified Likert scale (“yes,” “no,” or “don’t know”), with positive responses (such as valuing dental check-ups) scored as 1. Practices included behaviors, such as brushing frequency and dental visits, with desirable practices scoring 1. Scores <6 points (<60%) for KAP were considered poor, while scores between 4 and 5 points (40%-50%) were classified as average. The influence of educational level and family income on KAP scores was also analyzed.

Data collection

Data were collected by administering the questionnaire to eligible parents or caregivers in the outpatient department of the Department of Pediatric and Preventive Dentistry at two dental colleges, Regional Dental College in Guwahati, Assam, and Dental College in Azamgarh, Uttar Pradesh, targeting mothers of children aged 0-6 years in the Kamrup (Metro) district of Assam, and the Azamgarh district of Uttar Pradesh. The principal investigator coordinated the study protocol, while trained faculty and postgraduate students from both colleges facilitated data collection at their respective sites. Regular virtual meetings and shared documentation ensured consistent methodology and data integrity across both centers. This collaborative approach enhanced the study’s sample diversity and increased its generalizability. The questionnaire was self-administered; however, assistance was provided to the participants with low literacy levels to ensure accurate responses.

Statistical analysis

Statistical analyses were performed using the Stata Statistical Software Release 18 (StataCorp LLC, College Station, TX, USA). Participants’ responses were evaluated using the chi-square goodness-of-fit test. Normality testing of the KAP scores using the Shapiro-Wilk test confirmed a normal distribution (p>0.05), justifying the use of parametric tests. One-way analysis of variance was used to compare maternal KAP scores across different independent variables. Pearson's correlation analysis was conducted to examine the relationship between KAP scores and independent variables. All statistical tests were performed with a significance threshold of p<0.05.

## Results

The descriptive analysis of the study population (n=300) revealed significant variations across different parameters. A total of 96 (32%) mothers were aged 26-30 years, with an insignificant age distribution difference (p=0.082). A round 209 (69.7%) mothers were graduates and 188 (62.7%) were professionals, with no significant differences in education (p=0.061) or occupation (p=0.141). Urban residents dominated, showing a significant rural-urban disparity (p=0.001). Income distribution was uneven but not significant (p=0.525). Approximately 143 (47.7%) mothers had dental visits in the last six months, but only 78 (54.5%) of those received treatment, with no significant differences (p=0.418 and p=0.180, respectively). The findings highlighted demographic disparities, particularly in age and residence (Table 1).

**Table 1:** Descriptive analysis of study population using chi-square goodness-of-fit test. *p<0.05 denotes statistical significance using the chi-square goodness-of-fit test. Data presented as frequency (n) and percentage (%), where n denotes the number of mothers as participants.

Parameters	Category	n	%	p-value
Mother's age group (years)	20-25	54	18.0	0.082*
	26-30	96	32.0	
	31-35	67	22.3	
	36-40	83	27.7	
Education	Below metric	35	11.7	0.061*
	10+2	56	18.7	
	Graduate	209	69.7	
Occupation	Housewife	112	37.3	0.141*
	Professional	188	62.7	
Residential status	Rural	74	24.7	0.001*
	Urban	226	75.3	
Income	<10K	45	15.0	0.525*
	10K-20K	89	29.7	
	20K-50K	108	36.0	
	50K-100K	46	15.3	
	>10K	12	4.0	
Any dental visits (in the last six months)	Yes	143	47.7	0.418*
	No	157	52.3	
Any dental treatment (in the last six months)	Yes	78	54.5	0.180*
	No	65	45.5	
Oral health awareness	Yes	234	78.0	0.001*
	No	64	22.0	

The survey revealed high awareness among mothers regarding key aspects of children’s oral health. A strong majority recognized the importance of brushing their child’s teeth twice daily, while 250 (83%) mothers knew that caries could affect children under two years. Most mothers correctly affirmed that a baby’s mouth should be cleaned after every feeding, even before tooth eruption, and 235 (78%) mothers acknowledged that spacing between teeth was normal in children. However, only 165 (55%) mothers were aware of the recommended weaning age from bottle to cup. In terms of attitude, 275 (92%) mothers agreed that a balanced diet is crucial for healthy tooth development, and 280 (93%) actively sought to improve their dental health knowledge. Positive practices were also observed, with 280 (93%) mothers offering plain water after feeding and 280 (93%) assisting their children in brushing. Despite this, only 85 (28%) mothers believed in pre-chewing food for their children, and only 15 (5%) mothers frequently gave sweet food. Overall, the knowledge and attitude scores were high, but gaps in awareness and practices were noted, particularly concerning feeding habits and preventive dental care. All responses were statistically significant (p<0.05, Table 2).

**Table 2:** Descriptive analysis of questionnaire for participant’s responses. *p<0.05 denotes statistical significance using the chi-square goodness-of-fit test. K1-K10 denotes questions assessing knowledge of mothers about oral health of their children, A1-A10 denotes questions about attitude of mothers toward oral health of their children, and P1-P10 denotes questions about practice. Data are presented as frequency (n) and percentage (%), where n denotes the number of mothers as participants.

Category	Questions	Total	Yes		No		Don’t know		p-value
		n	n	%	n	%	n	%	
K1	Caries can affect children below 2 years of age	300	250	83	25	8	25	8	0.001*
K2	A complete set of baby teeth appears in the child’s mouth by the age of 24 months	300	180	60	80	27	40	13	0.001*
K3	Bakery products can cause tooth decay	300	175	58	50	17	75	25	0.001*
K4	The first baby tooth appears in the child’s mouth by six months of age	300	190	63	90	30	20	7	0.001*
K5	Should a baby’s mouth be cleaned after every feed, even before teeth erupt?	300	230	77	45	15	25	8	0.001*
K6	Brushing your baby’s teeth twice daily is important for preventing caries in your child’s mouth	300	290	97	5	2	5	2	0.001*
K7	You should start using fluoridated toothpaste to brush your child’s teeth after three years of age	300	185	62	45	15	70	23	0.001*
K8	Weaning from a baby bottle to a sipping cup should start at around six months of age	300	165	55	45	15	90	30	0.001*
K9	Can improper brushing lead to gum disease?	300	265	88	10	3	25	8	0.001*
K10	Is the spacing between teeth normal in children?	300	235	78	35	12	30	10	0.001*
A1	Do you believe that sharing feeding utensils (e.g., spoons) can transmit bacteria causing tooth decay?	300	150	50	95	32	55	18	0.001*
A2	Do you agree that a balanced diet is crucial for healthy tooth development in babies?	300	275	92	5	2	20	7	0.001*
A3	Do you think nighttime bottle or breastfeeding can contribute to tooth decay?	300	215	72	25	8	60	20	0.001*
A4	Should a child’s first dental visit occur by the age of one?	300	220	73	30	10	50	17	0.001*
A5	Do you agree that frequent or prolonged breastfeeding/bottle feeding can cause tooth decay?	300	170	57	80	27	50	17	0.001*
A6	Do you actively seek ways to improve your knowledge about dental health?	300	280	93	15	5	5	2	0.001*
A7	Do you think a child can effectively clean and brush their teeth without assistance?	300	170	57	105	35	25	8	0.001*
A8	Is swallowing toothpaste harmful to a child’s health?	300	220	73	50	17	30	10	0.001*
A9	Do you believe prolonged pacifier use can affect normal teeth development in children?	300	155	52	60	20	85	28	0.001*
A10	Should parents bite food into small pieces before feeding it to their child?	300	85	28	200	67	15	5	0.001*
P1	Do you frequently give sweet foods to children throughout the day?	300	15	5	260	87	25	8	0.001*
P2	Do you often provide sweetened juice in a bottle to children?	300	30	10	250	83	20	7	0.001*
P3	Do you offer plain water to your child after each feed?	300	280	93	15	5	5	2	0.001*
P4	Do you take your child for a dental check-up every six months?	300	235	78	25	8	40	13	0.001*
P5	Do you clean your baby’s gums daily with a clean gauze/cloth?	300	240	80	45	15	15	5	0.001*
P6	Do you use a pea-sized amount of toothpaste when brushing your child’s teeth?	300	225	75	50	17	25	8	0.001*
P7	Do you dip a pacifier in sweet liquids for your child?	300	95	32	125	42	80	27	0.001*
P8	Do you assist your child in brushing and cleaning their teeth?	300	280	93	20	7	0	0	0.002*
P9	Do you give teething rings to your baby during teething?	300	135	45	75	25	90	30	0.001*
P10	Do you provide fibrous fruits to children as snacks between meals?	300	210	70	45	15	45	15	0.001*

The study revealed significant differences in KAP scores across the mothers’ educational levels (p<0.05). Graduate mothers scored highest in knowledge (8.34±1.23), attitude (6.95±1.12), and practice (6.59±1.15), showing good KAP scores, followed by 10+2 educated mothers. The below-metric mothers had the lowest scores (5.16±0.82, 4.87±0.75, 3.62±0.74), showing poor KAP scores. The statistically significant differences between KAP scores suggest that maternal education directly influences oral health awareness and practices. Higher education correlated with better KAP, highlighting the need for targeted interventions for less-educated mothers to improve their children’s oral care (Table 3).

**Table 3:** Comparison of knowledge, attitude and practice (KAP) scores between different educational subgroups of mothers with one-way analysis of variance (ANOVA) test. *p<0.05 denotes statistical significance using a one-way ANOVA test. n denotes the number of mothers as participants, and KAP scores are presented as mean±standard deviation. Below metric: Education below 10th grade/secondary level; 10+2: Higher secondary education; Graduate: Completed a bachelor's degree program.

Education level of mothers		Below metric	10+2	Graduate	p-value
Participants	n (%)	45 (15.0)	115 (38.3)	140 (46.7)	
Knowledge score	Mean±SD	5.16±0.82	6.66±1.16	8.34±1.23	0.002*
Attitude score	Mean±SD	4.87±0.75	6.50±1.08	6.95±1.12	0.001*
Practice score	Mean±SD	3.62±0.74	5.73±1.02	6.59±1.15	0.001*

The study revealed a strong positive correlation between family income and the KAP scores (p<0.001). Mothers in the highest income bracket (> Rs. 100k) demonstrated significantly better knowledge (8.23±1.25), attitude (8.16±1.04), and practice scores (8.34±1.08) compared to those earning below Rs. 10k (4.23±0.85, 4.78±0.72, 3.65±0.65, respectively, for KAP scores). The progressive improvement across income groups was most pronounced in practice scores, showing a 129% difference between extreme groups. These findings suggested that economic status significantly influenced oral health awareness and behavior (Table 4).

**Table 4:** Comparison of knowledge, attitude and practice (KAP) scores between different income subgroups with one-way analysis of variance (ANOVA) test. *p<0.05 denotes statistical significance using a one-way ANOVA test. n denotes the number of mothers as participants, and KAP scores are presented as mean±standard deviation.

Income/month		Below Rs. 10k	Rs. 10k to Rs. 20k	Rs. 20k to Rs. 50k	Rs. 50k to Rs. 100k	Above Rs. 100k	p-value
Participants	n (%)	62 (20.7)	82 (27.3)	72 (24.0)	52 (17.3)	32 (10.7)	
Knowledge score	Mean±SD	4.23±0.85	5.87±1.13	6.64±1.25	8.12±1.14	8.23±1.25	0.001*
Attitude score	Mean±SD	4.78±0.72	5.65±0.98	6.43±1.02	7.86±1.12	8.16±1.04	0.001*
Practice score	Mean±SD	3.65±0.65	4.68±0.95	5.96±1.08	7.45±1.12	8.34±1.08	0.001*

The correlation analysis indicated that maternal education and income positively influenced knowledge and practice scores, with education having a stronger impact on practice (r=0.65). Oral health education had the most substantial effect, particularly in practice (r=0.78). Dental visits were strongly correlated with practice (r=0.72) but not with knowledge or attitudes, while maternal age had no significant impact on any KAP domain (Table 5).

**Table 5:** Pearson's correlation of knowledge, attitude and practice score with independent variables of mother. *p<0.05 denotes statistical significance using Pearson's correlation test. r=0 denotes no correlation, 0<r≤0.3 denotes weak correlation, 0.3<r≤0.6 denotes moderate correlation, and r>0.6 denotes strong correlation.

Parameters	Knowledge		Attitude		Practice	
	r value	p-value	r value	p-value	r value	p-value
Age	-0.12	0.135	-0.09	0.241	0.15	0.426*
Education	0.52	0.023*	0.35	0.329	0.65	0.016*
Income	0.38	0.031*	0.28	0.042*	0.52	0.022*
Dental visits	0.42	0.116	0.45	0.193	0.72	0.046*
Oral health education	0.62	0.023*	0.54	0.031*	0.78	0.018*

## Discussion

The findings of our study revealed that most mothers had a solid understanding of the key oral health concepts. For instance, most of the mothers knew that brushing a child’s teeth twice daily is crucial for preventing caries, and were aware that caries can strike children under two years old. Similar findings were reported by Alkhtib and Morawala [13], who assessed the KAP scores of mothers of preschool children in Qatar and reported that most of the mothers felt that their children should brush their teeth before three years of age. However, our study also found gaps in knowledge, such as only a few mothers knew that weaning from a bottle to a sipping cup should start around six months. This aligns with a previous study in Karnataka, where few parents were aware of proper weaning timelines, and the mean knowledge score of parents for early childhood caries was low, highlighting a common blind spot in early dental-care education [14].

Regarding attitudes, the mothers in our study showed strong commitment to their children’s oral health. Most of the mothers agreed that a balanced diet is vital for healthy teeth, and said they actively seek ways to learn more about dental health. This enthusiasm is a significant foundation for developing better habits. A study by Chhabra and Chhabra [15] in India found similar positive attitudes, with mothers valuing regular dental check-ups. However, our study showed some hesitation in certain areas, such as a few mothers believed that sharing utensils could spread bacteria that cause tooth decay. This contrasts with a study by Ashkanani and Al-Sane [16] in Kuwait, in which most of the parents acknowledged this risk, suggesting cultural or educational differences in how oral health risks are perceived.

The practice section of our study revealed both strengths and areas for improvement. On the positive side, most of the mothers reported offering plain water after feeding and assisting their children with brushing habits that could significantly reduce the caries risk. These findings contrast with those of a previous study in Kuwait, where parents had weak knowledge and practice regarding their children’s oral health [17]. However, in our study, few mothers believed in pre-chewing food for their children, a practice that can transmit harmful bacteria, and very few admitted to frequent consumption of sweet foods. While the low rate of sweet food consumption is encouraging, the pre-chewing habit suggests a need for targeted education [18].

One of the most striking findings was how strongly education and income shaped KAP scores. Mothers with graduate-level education scored highest in knowledge (8.34±1.23), attitudes (6.95±1.12), and practices (6.59±1.15), while those with less than a high school education scored lowest (5.16±0.82, 4.87±0.75, 3.62±0.74). This gap underscores how education empowers parents to make informed choices about their children’s health. A similar pattern was observed in a study by Tamannur et al. [19], who reported that the oral hygiene of children was significantly associated with family income and the mother's educational status. Our correlation analysis further showed that education had a moderate to strong impact on practice scores (r=0.65), reinforcing its role in behavioral change.

In our study, mothers in the highest income bracket had significantly better KAP scores than those with low earnings, with a 129% difference in practice scores between the extremes. This finding suggests that financial resources provide access to better education, dental care, and healthier products. Nota et al. [20] found a significant association between parents’ status and children’s oral hygiene. Similarly, Ellakany et al. [21] found a significant association between mothers’ age, education, income, and oral hygiene of their children.

Oral health education stood out as a game changer, showing the strongest correlation with the practice scores (r=0.78). This suggests that targeted programs can bridge gaps, especially for less educated or lower-income mothers. A systematic review by Stein et al. [22] reported that education programs are effective in preventing plaque accumulation, but not dental caries, in school children. Similarly, our finding that dental visits correlated strongly with practice (r=0.72) but not with knowledge or attitudes highlights the practical impact of professional guidance.

Interestingly, maternal age showed no significant correlation with KAP scores, suggesting that experience alone does not drive better oral health behaviors. This finding contrasts with a study by Wigen and Wang [23] in Norway, in which older parents had slightly higher knowledge scores (r=0.28). This difference might reflect cultural contexts, where younger mothers in India may have more access to modern health information through the media or community programs.

Our study revealed that despite high maternal knowledge, optimal oral health practices were not universally adopted, likely due to cultural practices such as pre-chewing food (reported by 28% of mothers) and access barriers, including limited availability of dental services or financial constraints in urban areas of Kamrup (Metro), Assam, and Azamgarh, Uttar Pradesh, which may hinder the translation of knowledge into consistent behaviors. 

These findings have several practical implications. High awareness among mothers is a strong starting point, but gaps in specific areas, such as weaning timelines or the risks of sharing utensils, require tailored education campaigns. The stark influence of education and income highlights the need for equitable access to resources, especially for marginalized families. Community-driven initiatives could serve as an effective model, delivering workshops in local languages to enhance parental knowledge and improve oral health practices for children. Schools and pediatric clinics can also play a role in integrating oral health tips into routine visits.

The strengths of this study include its robust methodology, well-validated questionnaire (Cronbach’s alpha=0.81), and sample size powered to detect differences. However, this study had some limitations. Hospital-based settings might not fully represent rural or non-hospital attending populations, and self-reported data could introduce bias. The reliance on self-reported data may introduce bias, as mothers might have provided socially desirable responses regarding practices such as assisting with brushing or limiting sweet foods, potentially overestimating positive behaviors and affecting the validity of the findings. This study also has potential sampling bias since only mothers of pediatric dental patients were surveyed, excluding parents from the general community. Their views may differ due to prior dental experiences or healthcare exposure, limiting the generalizability of findings to broader populations. Future research should include more diverse parent samples. The study primarily recruited participants from urban areas within the Kamrup (Metro) district of Assam and the Azamgarh district of Uttar Pradesh, as data were collected at the OPDs of Regional Dental College, Guwahati, and Dental College, Azamgarh, both located in urban settings. Therefore, this limits the generalizability of the study. Future research could explore longitudinal designs to track how KAP changes over time, or include fathers to capture a broader family perspective in a larger, more diversified sample.

## Conclusions

In summary, this study highlighted that mothers of young children demonstrated commendable levels of knowledge and positive attitudes toward oral health, especially concerning brushing habits and the importance of a balanced diet. However, certain misconceptions and inconsistent practices, such as delayed weaning and pre-chewing food, indicated areas that required further education. The data clearly showed that maternal education and family income played key roles in shaping oral health knowledge, beliefs, and behaviors. Mothers with higher educational qualifications and better economic status exhibited significantly stronger performance across all domains. Importantly, the findings underscored the transformative role of oral health education and routine dental visits in promoting better preventive practices. Despite the high baseline awareness observed, there remained a pressing need to address disparities and strengthen community outreach. Educational programs delivered through local platforms in regional languages are found to be effective in bridging knowledge gaps, correcting harmful practices, and empowering mothers to make informed decisions about their children's oral health. These insights are crucial for informing public health strategies focused on early intervention and reducing the burden of oral diseases in young children. To address identified knowledge gaps and socioeconomic disparities, policymakers should integrate oral health counseling into prenatal and antenatal care programs and implement government-led community workshops in local languages to promote equitable oral health practices and reduce childhood oral disease prevalence.

## Appendices

**Table 6:** Demographic details (in English language). Credit: Pranamee Barua.

REGIONAL DENTAL COLLEGE, GUWAHATI Department of Pedodontics Questionnaire		
Demographic details		
Child name		
Child age		
Child gender		
Mother name		
Mother age		
Education	Below metric	
	10+2	
	Graduate	
Occupation	Housewife	
	Professional	
Residential status	Rural	
	Urban	
Income	<10000	
	10000-20000	
	20000-50000	
	50000-100000	
	>100000	
Any dental visits (in last 6 months)	Yes	
	No	
Any dental treatment (in last 6 months)	Yes	
	No	

**Table 7:** Questions for assessment of knowledge, attitude and practice of oral health in children (in English language). Credit: Pranamee Barua.

AWARENESS ABOUT CARIES *Tick the most appropriate answer. Tick only one answer (Yes/No or Don’t know)				
S.no	Question	Yes	No	Don’t know
K1	Caries can affect children below 2 years of age			
K2	Complete set of baby teeth appear in the child’s mouth by the age of 24months			
K3	Bakery products can cause tooth decay			
K4	The first baby tooth appear in the child’s mouth by 6months of age			
K5	Should a baby’s mouth be cleaned after every feed even before teeth erupt?			
K6	Brushing baby’s teeth twice daily is important for preventing caries in your child’s mouth			
K7	You should start using fluoridated toothpaste to brush your child’s teeth after 3years of age			
K8	Weaning from a baby bottle to a sipping cup should start at around 6months of age			
K9	Can improper brushing lead to gum disease?			
K10	Is spacing between teeth normal in children?			
A1	Do you believe that sharing feeding utensils (e.g., spoons) can transmit bacteria causing tooth decay?			
A2	Do you agree that a balanced diet is crucial for healthy tooth development in babies?			
A3	Do you think nighttime bottle or breastfeeding can contribute to tooth decay?			
A4	Should a child’s first dental visit occur by the age of one?			
A5	Do you agree that frequent or prolonged breastfeeding/bottle feeding can cause tooth decay?			
A6	Do you actively seek ways to improve your knowledge about dental health?			
A7	Do you think a child can effectively clean and brush their teeth without assistance?			
A8	Is swallowing toothpaste harmful to a child’s health?			
A9	Do you believe prolonged pacifier use can affect normal teeth development in children?			
A10	Should parents bite food into small pieces before feeding it to their child?			
P1	Do you frequently give sweet foods to children throughout the day?			
P2	Do you often provide sweetened juice in a bottle to children?			
P3	Do you offer plain water to your child after each feed?			
P4	Do you take your child for a dental check-up every six months?			
P5	Do you clean your baby’s gums daily with a clean gauze/cloth?			
P6	Do you use a pea-sized amount of toothpaste when brushing your child’s teeth?			
P7	Do you dip a pacifier in sweet liquids for your child?			
P8	Do you assist your child in brushing and cleaning their teeth?			
P9	Do you give teething rings to your baby during teething?			
P10	Do you provide fibrous fruits to children as snacks between meals?			
